# PANCREATODUODENECTOMY FOR SOLID PSEUDOPAPILLARY TUMOR OF THE PANCREAS: A MULTI-INSTITUTION STUDY

**DOI:** 10.1590/0102-672020190001e1442

**Published:** 2019-08-26

**Authors:** Orlando Jorge M TORRES, Marcelo Bruno de REZENDE, Fábio Luiz WAECHTER, Romerito Fonseca NEIVA, José Maria A MORAES-JUNIOR, Camila Cristina S TORRES, Eduardo de Souza M FERNANDES

**Affiliations:** 1 Department of Gastrointestinal Surgery, Hepatopancreatobiliary Unit, Universidade Federal do Maranhão, São Luiz, MA;; 2 Department of Gastrointestinal and Transplant Surgery, Hospital Israelita Albert Einstein, São Paulo, SP;; 3 Department of Gastrointestinal Surgery, Hepatopancreatobiliary Unit, Santa Casa Hospital, Porto Alegre, RS;; 4 Department of Gastrointestinal Surgery, Hepatopancreatobiliary and Transplant Unit, Universidade Federal do Rio de Janeiro, RJ, Brazil.

**Keywords:** Solid pseudopapillary tumor, Solid pseudopapillary neoplasm, Frantz’ tumor, Pancreatectoduodenectomy, Surgical outcomes, Tumor sólido pseudopapilar, Neoplasia sólida pseudopapilar, Tumor de Frantz, Duodenopancreatectomia, Resultados cirúrgicos

## Abstract

*****Background***:**

Solid pseudopapillary tumor of the pancreas is a rare low-grade malignant neoplasm. Most patients present with nonspecific symptoms until the tumor becomes large. Complete surgical resection by pancreatoduodenectomy is the treatment of choice for tumors located in the head of the pancreas

*****Aim***:**

To analyzed the clinicopathologic features, management, and outcomes of patients who had solid pseudopapillary tumor of the head pancreas and underwent surgical resection.

*****Methods***:**

Were analyzed 16 patients who underwent pancreatoduodenectomy for this condition.

*****Results***:**

Mean age was 25.7 years old, and 15 patients were female (93.7%). Nonspecific abdominal pain was present in 14 (87.5%). All underwent computed tomography and/or magnetic resonance imaging as part of diagnostic workup. The median diameter of the tumor was 6.28 cm, and surgical resection was performed with open or laparoscopic pancreatoduodenectomy without neoadjuvant chemotherapy. Postoperative complications occurred in six patients (37.5%) and included pancreatic fistula without mortality. The mean of hospital stay was 10.3 days. Median follow-up was 3.6 years, and no patient had local recurrence or metastatic disease.

*****Conclusion***:**

For these patients surgical resection with pancreatoduodenectomy is the treatment of choice showing low morbidity, no mortality, and good long-term survival.

## RESUMO

*****Racional***:**

Tumor sólido pseudopapilar do pâncreas é neoplasia maligna rara, de baixo grau de malignidade. A maioria dos pacientes apresenta sintomas inespecíficos até que o tumor aumente de tamanho. A ressecção cirúrgica completa através a duodenopancreatectomia é o tratamento de escolha para os localizados na cabeça do pâncreas.

***Objetivo*:**

Analisar as características clinicopatológicas, tratamento e resultados de pacientes com tumor sólido pseudopapilar do pâncreas localizado na cabeça do pâncreas submetidos à ressecção cirúrgica.

*****Método***:**

Foram analisados 16 pacientes com duodenopancreatectomia devido a esse tumor localizado na cabeça do pâncreas.

Havia 15 mulheres (93,7%) e a média de idade era de 25,7 anos. Dor abdominal não específica esteve presente em 14 pacientes (87,5%). Todos realizaram tomografia computadorizada do abdome e/ou ressonância nuclear magnética como parte da investigação. O diâmetro médio do tumor era de 6,28 cm e a ressecção cirúrgica foi realizada por duodenopancreatectomia, tanto por laparotomia quanto por videolaparoscopia, com ou sem quimioterapia neoadjuvante. As complicações pós-operatórias ocorreram em seis pacientes (37,5%) e incluíram fístula pancreática, sem mortalidade. O tempo médio de internação hospitalar foi de 10,3 dias. O tempo médio de seguimento foi de 3,6 anos e nenhum paciente apresentou recorrência local ou doença metastática.

***Conclusões*:**

A ressecção cirúrgica através da duodenopancreatectomia é o tratamento de escolha para estes pacientes. Os resultados mostraram baixa morbidade, nenhuma mortalidade e boa sobrevida em longo prazo.

## INTRODUCTION

Solid pseudopapillary tumor (SPT) of the pancreas, first described by Virginia K. Frantz in 1959, is a rare, low-grade malignant tumor of the exocrine pancreas. The tumor is also called Frantz tumor of the pancreas. More than 90% of patients are young females, suggesting some association with female hormones. This tumor accounts for approximately 1-2.5% of all exocrine pancreatic neoplasms, and most are large tumors that are confined to the pancreas, located in the head (26-34%) and the body and tail of the pancreas (66-74%)^11^ ^,^ ^15^ ^,^ ^16^. 

The World Health Organization (2010) defined the tumor as an epithelial low-grade malignant neoplasm with a gross pseudopapillary appearance and a cystic microscopic appearance. The risk of metastasis to the liver and peritoneum is low (10-15%), and long-term survival has been reported after surgical resection^9^ ^,^ ^14^. 

Most of the patients are asymptomatic or presented with nonspecific symptoms until the tumor became large. Lesions in the pancreatic head are more likely to cause early symptoms, and common findings include upper abdominal discomfort, anorexia, postprandial fullness, weight loss, and a palpable mass on physical examination. Tumors located in the pancreatic head are more likely to cause symptoms, even if they are small. Hemoperitoneum secondary to rupture of the tumor may occur as an emergency situation^5^ ^,^ ^7^ ^,^ ^20^.

Computed tomography or magnetic resonance imaging of SPT of the pancreas may show an encapsulated mass with solid and cystic components. Hemorrhage or cystic degeneration also may be observed. Complete surgical resection is the treatment of choice, and the approach is dependent on the location of disease. For patients with tumor located in the head of the pancreas, pancreatoduodenectomy is associated with an overall good prognosis, although recurrence can occur^13^ ^,^ ^15^ ^,^ ^21^. 

This study aimed to analyze the clinicopathologic features, management, and outcomes of patients with Frantz tumor of the pancreas who underwent pancreatoduodenectomy.

## METHODS

We performed a retrospective analysis of 16 patients who underwent pancreatoduodenectomy for SPT of the pancreas (Frantz tumor) at four hepatopancreatobiliary centers in Brazil between July 2001 and February 2018. Data obtained from the clinical records included demographic features, clinical presentation, and radiologic findings as well as a description of the surgical procedure, postoperative course, and follow-up. Most patients underwent tumor marker (CEA and CA 19-9) assessment and histopathologic analysis. Ethical approval was not required and patient identifying knowledge was not presented in this report. 

Follow-up occurred in an outpatient setting and included clinical evaluation, laboratory tests, and computed tomography scan at 1 month, 3 months, and then every 12 months. Postoperative pancreatic fistula was defined according to the International Study Group of Pancreatic Surgery (ISGPS) definition, and the Clavien-Dindo classification was used to define postoperative complications^2^ ^,^ ^10^.

## RESULTS

**FIGURE 1:**
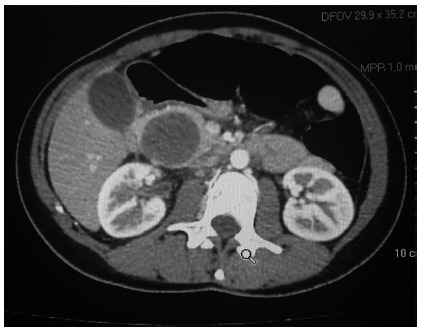
Computed tomography in patient with solid pseudopapillary tumor of the pancreas

The clinicopathologic characteristics and outcome are summarized in Table 1. Mean age was 25.7 years old (11-51), and 15 were female (93.7%). Abdominal pain was present in 14 patients (87.5%), and other clinical manifestations were nausea, vomiting, and left upper quadrant mass. A palpable abdominal mass was observed in two (12.5%). Median duration of symptoms was three weeks (four days to five months). All patients underwent computed tomography and/or magnetic resonance imaging as part of the diagnostic workup (Figure 1). In all patients, computed tomography showed a cystic solid mass or a clear-bordered cystic mass with heterogeneous density. Eight patients (50.0%) underwent magnetic resonance imaging, and hyperintensity with heterogeneity of the tumor on T1-weighted images with a higher signal on T2 was observed. The tumor was located in the head of the pancreas in all patients (100%), and the median diameter was 6.28 cm (3.5-10.7). Assessment for tumor markers CEA and CA 19-9 was performed in 11 (68.7%) patients, and findings were normal. 

**TABLE 1:** Clinicopathological characteristics of the patients

	Year	Gender	Age	Symptoms	Image	Approach	Size (cm)	Laparoscopy	Anastomosis	LoS (day)	Complication	Recurrence
1	2001	F	19	Abdominal pain	CT	Whipple	4,0	No	PJ	17	Fistula A	No
2	2005	F	19	Abdominal pain	CT	Whipple	6,3	No	PG	8	No	No
3	2007	F	16	Abdominal pain	MRI	PPPD	7.5	No	PJ	18	Fistula B	No
4	2008	F	28	Abdominal pain	CT	PPPD	5.2	No	PJ	13	Infected collection	No
5	2012	F	32	Abdominal pain	CT/MRI	SSPPD	6,6	No	PJ	14	Fistula A	No
6	2014	F	43	Abdominal pain	MRI	PPPD	8.4	No	PG	11	No	No
7	2014	F	18	Abdominal pain	CT	SSPPD	3.5	Yes	PJ	5	No	No
8	2015	F	32	Nausea, vomiting, weight loss	CT	SSPPD	8.0	No	PJ	14	Bleeding	No
9	2015	F	17	Abdominal pain	MRI	SSPPD	3.9	Yes	PJ	6	No	No
10	2016	M	48	Abdominal pain	MRI	PPPD	4.1	No	PG	16	Pancreatitis	No
11	2016	F	17	Abdominal pain, upper quadrant abdominal mass	CT	Whipple	10.0	No	PJ	8	No	No
12	2017	F	15	Abdominal pain	CT	Whipple	9.0	No	PJ	7	No	No
13	2017	F	51	Abdominal pain	CT/MRI	SSPPD	3,8	No	PJ	¨6	No	No
14	2017	F	11	Abdominal Pain	MRI	PPPD	5.6	No	PJ	9	No	No
15	2018	F	32	Nausea, weight loss, upper quadrant abdominal mass	CT	Whipple	10.7	No	PJ	7	No	No
16	2018	F	14	Abdominal pain	CT/MRI	SSPPD	4.0	Yes	PJ	6	No	No

F=female; M=male; cm=centimeters; LoS=length of stay; CT=computed tomography; MRI=magnetic resonance image; PJ=pancreatojejunostomy; PG=pancreatogastrostomy; PPPD=pylorus-preserving pancreatoduodenectomy; SSPPD=subtotal stomach preserving pancreatoduodenectomy

**FIGURE 2:**
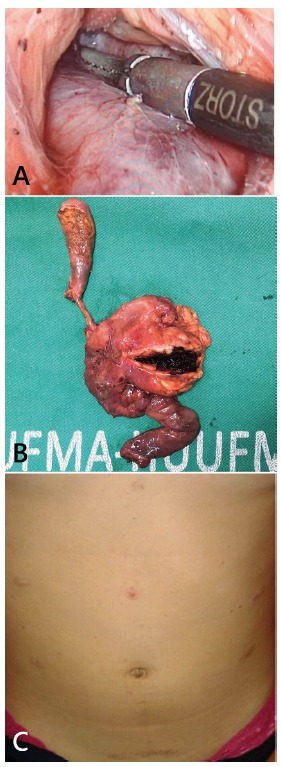
A) Laparoscopic pancreatoduodenectomy; B) specimen; C) final aspect of the abdomen

Surgical resection was performed in all patients, without neoadjuvant chemotherapy. Pancreatoduodenectomy was the procedure of choice in all patients (100.0%), and three different approaches (classic Whipple, pylorus-preserving, and stomach-preserving) were used. Three patients (18.7%) underwent pancreatoduodenectomy with laparoscopy (Figure 2). No liver metastasis was observed. The mean of hospital stay was 10.3 days (5-18). Postoperative complications (2 IIIa and 1 IIIb - Clavien-Dindo) were observed in six (37.5%; grade A or B pancreatic fistula in three patients; infection, bleeding, and pancreatitis in one patient each). Patient 3 had a grade B pancreatic fistula that was treated with percutaneous drainage guided by computed tomography; patient 4 had an infection that was drained percutaneously with ultrasonography; patient 8 had hypovolemia as a result of abdominal bleeding and underwent surgical intervention; patient 10 had acute pancreatitis that resolved after clinical treatment. No hospital mortality was observed. All patients were followed by a clinical oncologist, and no adjuvant chemotherapy was administered. One was lost to follow-up (case 1) after one year without recurrence. Median follow-up was 3.6 years (nine months to five years), and no patient had local recurrence or metastatic disease. 

**FIGURE 3:**
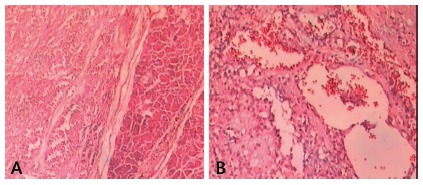
Microscopic characteristics of solid pseudopapillary tumor of the pancreas: A) HE 40x; B) HE 100x

The gross appearance of the specimen showed mixed solid and cystic components in encapsulated neoplasm. The cut surface had areas of necrosis and hemorrhage with solid and cystic patterns. In all patients the resection margins were free of tumor. The histopathologic features showed a pseudopapillary pattern, cystic degeneration, cytoplasmic granules, and hemorrhage (Figure 3). No patient had vascular or perineural invasion, although patient 10 (6.25%) showed capsular invasion. No lymph node involvement was observed.

## DISCUSSION

Because of its low incidence, SPT of the pancreas is relatively unknown. The World Health Organization described two major histologic components of the tumor: solid and papillary. This low-grade malignant tumor is most frequently observed in young women, and the prognosis is favorable after complete resection, with approximately 80% of patients experiencing long-term survival. The current study included 15 cases (93.7%) in young women, confirming that SPN is most common in female younger than 35 years old. The relationship between SPT and sex hormones in women has been studied, and accelerated growth during pregnancy and the influence of estrogenic molecules on the proliferation of SPT cells has been observed. Others believe that embryonic development of SPT may derive from migrated primordial ovarian cells. More aggressive and rapidly fatal evolution of SPT is more common in male. In the current study, the mean age was 25.7 years old (11-51)^1^ ^,^ ^15^ ^,^ ^16^ ^,^ ^20^.

Solid pseudopapillary tumor of the pancreas can involve any part of the organ, but this study included only patients with tumor located in the head. Symptoms are usually non-specific, and abdominal pain, abdominal mass, and incidental findings are observed in most cases. In the current study, all patients had symptoms. Abdominal pain was the most common symptom, observed in 13 (81.2%), but nausea, vomiting, weight loss, and upper right quadrant abdominal mass were also observed. Jaundice has not been described as a common symptom^14^ ^,^ ^15^ ^,^ ^16^. 

The diagnosis of SPT is based on the findings of computed tomography and magnetic resonance imaging. On computed tomography, the tumor appears as a solid or cystic large mass, with peripheral arterial enhancement, circumscribed by a capsule with central calcifications. Cystic degeneration and calcifications are often identified within the tumor. In the case of a non-cystic mass, SPT may resemble a mucinous cystadenocarcinoma or a non-functioning pancreatic endocrine tumor. Enhancement in the arterial phase is typically seen in neuroendocrine tumors, and hypoattenuation is observed in the venous phase of adenocarcinomas. In the current study, abdominal computed tomography was the most common preoperative imaging study^7^ ^,^ ^14^ ^,^ ^15^ ^,^ ^20^.

 In some studies, preoperative diagnosis with percutaneous ultrasound-guided fine-needle aspiration has been performed. In the current study, preoperative cytologic diagnosis was not performed and solid cystic tumor was the most common radiographic finding. Serum tumor markers CEA and CA 19-9 do not provide useful diagnostic or prognostic information in patients with SPT because the levels are normal in all patients^7^ ^,^ ^14^ ^,^ ^15^ ^,^ ^20^. 

Histologic evaluation of this tumor shows a well-vascularized fibrous capsule and a mass that is completely solid or that has cystic components. They usually present with a pseudopapillary pattern with cystic degeneration or hemorrhage but rare mitotic figures. The most reliable marker to differentiate SPT and other pancreatic tumors is nuclear stabilization of β-catenin associated with a lack of membrane staining for E-cadherin. In some studies, serum tumor markers with percutaneous or endoscopic biopsy have been suggested to improve the accuracy of preoperative diagnosis. In the current study, serum tumor markers were measured in 11 (68.7%) patients, and findings were normal. In some cases, preoperative diagnosis may be difficult, and one third of patients may present with another histopathologic type of pancreatic neoplasm^5^ ^,^ ^7^ ^,^ ^13^ ^,^ ^20^. 

Surgical resection is the only curative treatment for SPT. Most series indicate that when a resectable tumor is discovered incidentally, resection results in long-term survival and provides a cure rate of up to 95%. Accurate diagnosis and differentiation from other more aggressive tumors is important^3^ ^,^ ^7^ ^,^ ^13^ ^,^ ^14^ ^,^ ^15^ ^,^ ^17^ ^,^ ^20^ ^,^ ^23^.

Pancreatoduodenectomy is the procedure of choice for patients with malignant tumors located in the head of the pancreas. Some complications are related to pancreatoduodenectomy, including pancreatic fistula, postoperative bleeding, delayed gastric empting, and infection. According to Callery et al.^6^, the main risk factors for pancreatic fistula are duct size of less than 3 mm, soft pancreas, pathology other than pancreatic adenocarcinoma, and intraoperative blood loss of more than 400 ml. In the current study, the fistula rate was 18.7% (n=3) because patients with SPT have a soft pancreas and duct size of less than 3 mm. Despite the 40% complication rate, no mortality was observed^5^ ^,^ ^6^ ^,^ ^7^ ^,^ ^13^. 

Laparoscopic pancreatoduodenectomy can be performed safely in patients with tumor located in the head of the pancreas. For patients with SPT, this procedure is safe and feasible, and the ones with it in pancreatic head are ideal candidates for laparoscopic or robotic pancreatoduodenectomy mainly young women with low-grade tumors, no vascular invasion, and no previous surgery or comorbidities ^3,4,8,12,18,19,21,22^. In the current study three (18.7%) young female with no history of surgery underwent laparoscopic surgical resection. No patient had regional lymph nodes or metastatic disease. Patient selection and experience with laparoscopic surgery are important factors for success. 

The role of chemotherapy for patients undergoing resection of SPT is under debate. Good results have been achieved with gemcitabine in patients with large tumors and uncertain behavior. A more aggressive course of treatment at presentation has been reported in patients with distant metastases or local recurrence^13^ ^,^ ^15^ ^,^ ^20^ ^,^ ^23^. 

Some studies have suggested that larger size, male, and younger age are associated with more aggressive disease and that these patients should be treated with a more radical procedure^5^ ^,^ ^7^. We performed pancreatoduodenectomy in just one male with a 4.1 cm tumor in 2016, with no recurrence so far. Because of the small number of patients in this series, it was difficult to identify the influence of these factors on malignant behavior. The survival rate after resection of SPT is high, and no malignant predictor has been found, such as tumor size or an infiltrative pattern. The risk of mortality is low, and the prognosis is usually favorable, with disease-free survival reported in more than 95% of patients^5^ ^,^ ^7^ ^,^ ^15^ ^,^ ^23^.

 In summary, solid pseudopapillary tumor of the pancreas is a rare, low-grade malignant tumor of the exocrine pancreas. These tumors occur more frequently in young female patients, suggesting that there may be some association with female hormones. Most tumors are large and are confined to the pancreas. In most young patients with abdominal symptoms, the diagnosis can be made with computed tomography or magnetic resonance imaging. Laparoscopic pancreatoduodenectomy can be performed in selected patients by surgeons with expertise in hepatopancreatobiliary and laparoscopic procedures. 

## CONCLUSION

Tumor in the head of the pancreas, surgical resection with pancreatoduodenectomy is the treatment of choice. This treatment usually results in low morbidity and good long-term survival. 
